# Accuracy of Computer-Assisted Template-Based Implant Placement Using Two Different Surgical Templates Designed with or without Metallic Sleeves: A Randomized Controlled Trial

**DOI:** 10.3390/dj7020041

**Published:** 2019-04-02

**Authors:** Marco Tallarico, Matteo Martinolli, Yong-Jin Kim, Fabio Cocchi, Silvio Mario Meloni, Adem Alushi, Erta Xhanari

**Affiliations:** 1 Department of Implantology and Prosthetic Aspects, Master of Science in Dentistry Program, Aldent University, 1001 Tirana, Albania; ertaxhanari@hotmail.com; 2 Private practice, 00143 Rome, Italy; 3 Private practice, 45014 Porto Viro, Italy; matteo.martinolli@hotmail.it; 4 Ilsan Apsun Dental Clinic, Ilsan 10381, Korea; ddskyj@naver.com; 5 Private practice, 41121 Modena, Italy; ing.fabiococchi@gmail.com; 6 Surgical, School of Dentistry, University of Sassari, 07100 Sassari, Italy; melonisilviomario@yahoo.it; 7 Department of Restorative Dentistry, University of Medicine Tirana, 1001 Tirana, Albania; info@ertaxhanari.com; 8 Private practice, 1001 Tirana, Albania

**Keywords:** intraoral scanner, digital impression, guided surgery, accuracy, dental implants

## Abstract

**Purpose:** To compare virtual planning accuracy of novel computer-assisted, template-based implant placement techniques, which make use of CAD/CAM stereolithographic surgical templates with or without metallic sleeves. Furthermore, to compare open versus closed sleeves for templates without metallic sleeves. **Materials and methods**: Any partially edentulous patients requiring at least one implant to be placed according to a computer-assisted template-based protocol were enrolled. Patients were randomized according to a parallel group design into two arms: Surgical template with or without metallic sleeves. Three deviation parameters (angular, horizontal, vertical) were defined to evaluate the discrepancy between the planned and placed implant positions. **Results**: No implants failed, and no complications were experienced. Forty-one implants were placed using surgical templates with metallic sleeves while 49 implants were placed with a surgical template without metallic sleeves. Of these, 16 implants were placed through open sleeves and 33 through closed sleeves. There was a statistically significant difference in angle (*p* = 0.0212) and in the vertical plan (*p* = 0.0073) with lower values for implants placed with a surgical template without metallic sleeves. In the test group, close sleeves were more accurate compared with open sleeves in angle (*p* = 0.0268) and in horizontal plan (*p* = 0.0477). Conclusion: With the limitations of the present study, surgical templates without metallic sleeves were more accurate in the vertical plan and angle compared to the conventional template with metallic sleeves. Open sleeves should be used with caution in the molar region only in case of reduced interarch space. Further research is needed to confirm these preliminary results.

## 1. Introduction

Nowadays, the development of three-dimensional (3D) imaging techniques and implant planning software has contributed to a large diffusion of prosthetically guided implant placement, nevertheless, the replacement of lost natural teeth is still a challenge for the clinician, mostly due to bone deficiency. Modern digital technology could help to improve patients’ acceptance and clinical success. In the last years, the use of digital intraoral optical scanner (IOS) has been shown to be a viable option for the rehabilitation of partial edentulous patients, even when associated to computer-guided template-assisted implant placement [1,2]. The clinician and the patient can benefit in terms of shorter treatment time if intraoral digital impression is used [2].

According to the Glossary of prosthodontic terms, surgical template (or surgical guide) is defined as ‘a guide used to assist in proper surgical placement and angulation of dental implants’ [3]. In a randomized controlled trial with five years of follow-up, the implant survival rate has been similar for conventional and computer-guided template-assisted implant placement procedures [4,5]. Furthermore, a significant reduction of post-operative pain and swelling, as well as statistically significant lower marginal bone loss were found five years after loading, placing implants with a template-based approach [5].

The main objective of the surgical guide is to guide implant drills and provide accurate implant placement according to the virtual, prosthetically driven, treatment plan. Thanks to improved technologies, including stereolithography, surgical templates are easy to produce, representing the union of guiding cylinders and contact surface. The latter fits either on hard and soft tissues giving stability, while, the cylinders work as a drill guides orienting the drill in the exact location and direction. Usually, surgical templates contain metallic sleeves to drive surgical drills. Recently, surgical templates without metallic sleeves have been designed and introduced in the dental market, with the goal to make guided surgery work-flow faster and easier. It is intrinsic that thanks to the absence of metal, the templates designed without metallic sleeve can be customized, requiring, for example, less mesio-distal space. Moreover, sleeve-designed templates can be produced with a slot (usually buccal or lingual located) that allows the horizontal insertion of the drills (open sleeve), reducing the minimum required interarch space, and also reducing the bone heating due to a direct saline irrigation on the drill. Another advantage of the digital work-flow and the development of high quality desktop 3D printers is making in-house surgical template production affordable [6]. This makes the sleeve-designed templates easier to produce and probably less expensive due to the absence of stainless steel or titanium drill-guiding tubes. Nevertheless, there is still lack of data about their accuracy and predictability. For all of these reasons, the possibility to easily customize the newly designed surgical templates should represent one of the most important benefits for the clinician. In addition, evidence that newly developed templates are better than conventional ones is still lacking.

The purpose of the present randomized controlled trial is to compare implant survival rate, complications, and accuracy of computer-assisted template-based implant placement using surgical templates designed with or without metallic sleeves. Furthermore, to compare implant accuracy using open or closed holes in case of tubeless templates. The null hypothesis was that there would be no differences between these groups. This trial is reported in accordance with the CONSORT (Consolidated Standards of Reporting Trials) statement (http://www.consort-statment.org) for improving the quality of reporting of parallel-group randomized trials.

## 2. Materials and Methods

This study was designed as a randomized controlled trial of parallel group design conducted at one center between May 2016 and March 2017. The study was performed after approval was received from the institutional review board of the Aldent University, Tirana, Albania (2/2017). The investigation was conducted according to the principles embodied in the Helsinki Declaration of 2013 and the trial was recorded and registered in the public register of clinical trials (www.clinicaltrials.gov) with the number NCT03641365. Surgical and prosthetic procedures were performed by one expert clinician (MT). All patients were informed about the nature of the treatment and their written consent was obtained. Data collection was designed to preserve patient anonymity.

Any partially edentulous patients aged 18 years or older, able to sign an informed consent, in need of an implant-supported fixed restoration was considered eligible for this study. Any potential implant location based on individual patient requirements was considered eligible in the present trial. Exclusion criteria were: General medical contraindication to oral surgery (American Society of Anesthesiologist, ASA, class III or IV); irradiation in the head and neck area less than one year before implantation; psychiatric problems; alcohol or drug abuse; pregnant or nursing; untreated periodontitis; severe bruxism or clenching; uncontrolled diabetes; poor oral hygiene and motivation; and inability to complete the follow-up.

**Figure 1:**
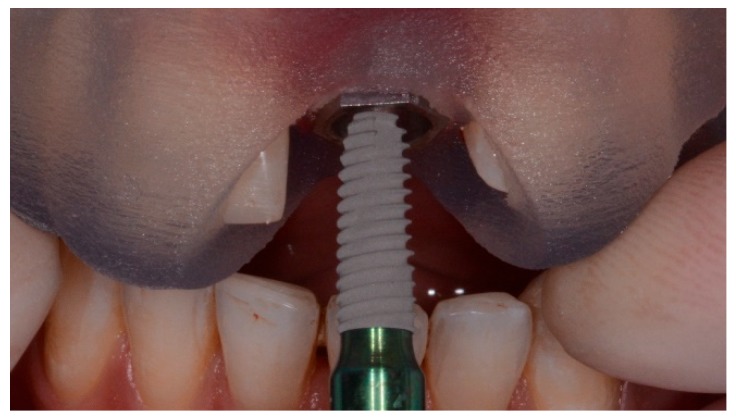
Surgical templates with metallic sleeves.

**Figure 2:**
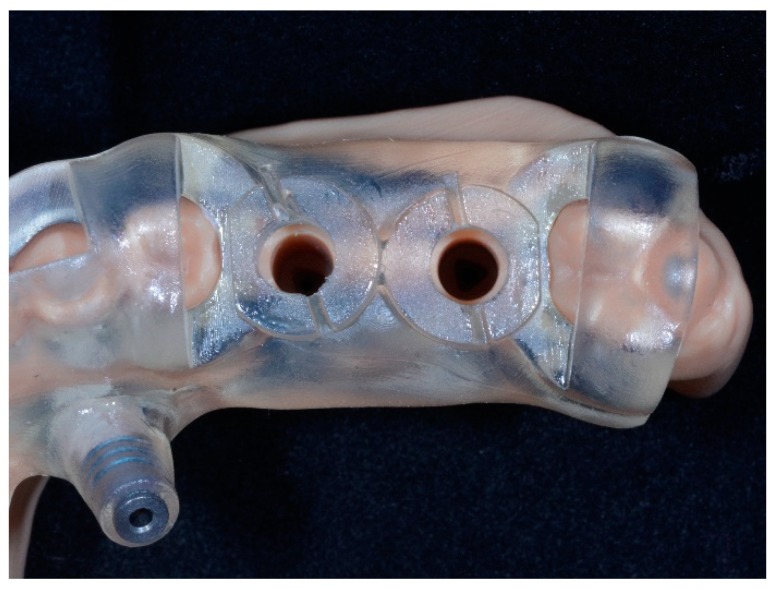
Surgical template without metallic sleeves: Closed sleeves.

**Figure 3:**
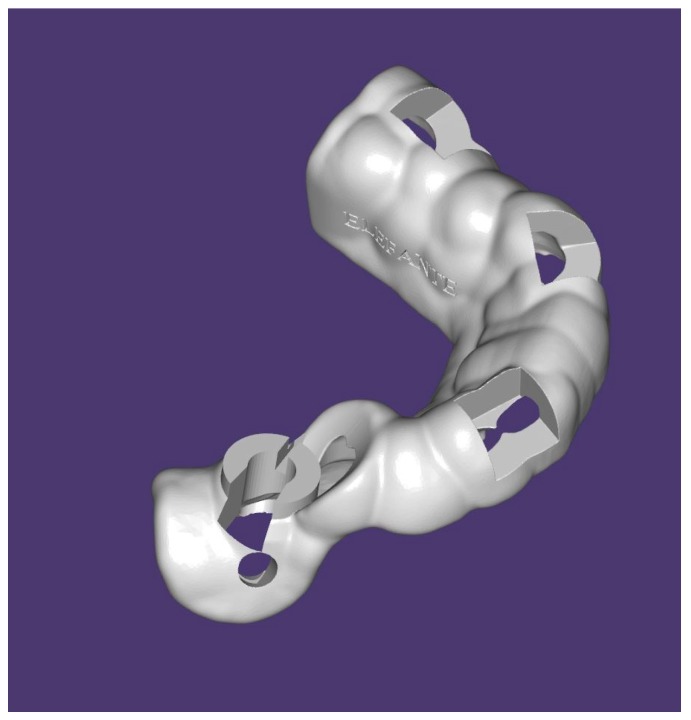
Surgical template without metallic sleeves: Open sleeves.

Enrolled patients received preoperative photographs, periapical radiographs or panoramic x-rays for initial screening and evaluation. The prosthetic-driven planning workflow started by taking a cone beam computed tomography (CBCT) scan (Cranex 3Dx, Soredex, Tuusula, Finland) by using a wax bite to separate dental arches. Then, the patients received intraoral digital impression taken using the 3M True Definition Scanner (3M Italia, Pioltello, Milano). The digital data (STL, Surface Tessellation Language) were imported in a 3D design software (exocad DentalCAD, Exocad GmbH, Darmstadt, Germany) to realize a virtual wax-up according to the functional and esthetic requirements. Then, the STL and DICOM (Digital Imaging and COmmunications in Medicine) data were imported in a 3D software planning program (3Diagnosys ver. 5.0, 3DIEMME srl, Cantù, Italy). Then, the reprocessed surface extrapolated from the DICOM data (by using a Hounsfield scale filter) and the surface generated by the master cast scanning process, or by the intraoral scanning process, were merged with the best-fitting repositioning tools of the software (3Diagnosys ver. 5.0, 3DIEMME srl). Afterwards, prosthetic-driven implants/abutments size and location were planned to take into account the bone quality and quantity, soft tissue thickness, anatomical landmarks, as well as type, volume, and shape of the final restoration. After careful functional and esthetic evaluation and final verification, the prosthetic-driven plan was approved. At this point, patients were randomly assigned to receive conventional stereolithographic surgical template with (control group, Figure 1) or without (test group, Figure 2 and Figure 3) metallic sleeves. Stereolithographic surgical templates were designed and fabricated by an independent certified center not previously involved in the study (New Ancorvis srl, Bargellino, Italy). In the test group, conventional templates with close-sleeve-design were produced to place implants between premolars. In case of implants to be placed in molar area, template with open sites were produced to solve interarch space limits.

All patients underwent professional oral hygiene, prophylactic antiseptic with 0.2% chlorhexidine (Curasept, Curaden Healthcare, Saronno, Italy) for one minute, and prophylactic antibiotic therapy (2 g of amoxicillin or clindamycin 600 mg if allergic to penicillin). Immediately before implant placement, the fit of the surgical templates was accurately tried in the patient mouth to achieve a stable fit (Fit Checker, GC—Tokyo, Japan). All patients were treated under local anesthesia using articaine with adrenaline 1:100000 administered 20 min before surgery. The surgical templates were stabilized on the residual teeth and fixed with two to three preplanned anchor pins.

Hopeless teeth were extracted at the end of the intervention in order to improve the stability of the surgical template and to provide more reference point for measurements of the implant accuracy. Nevertheless, in the case of immediate post-extractive implants, residual teeth were extracted as a-traumatically as possible immediately before surgery. In the test group, planned implants (Osstem TSIII, Osstem, Seoul, South Korea) were placed flapless using dedicate drills (OneGuide Kit, Osstem) in combination with a sleeve-designed surgical guide. In this case the drills were used directly through the sleeve-designed template without metallic tubs and without drill reductions. In the control group, planned implants (Osstem TSIII, Osstem, Seoul, South Korea) were placed flapless using dedicate drills (OsstemGuide Kit [Taper], Osstem) in combination with reduction tools, within the surgical templates containing metallic sleeves. If the keratinized gingiva and the amount of bone were adequate, the implants were placed flapless. Otherwise, a flap was elevated and then the wound was closed with single-stitch sutures using 4.0 resorbable suture material (Vicryl, Ethicon J&J International, Sint-Stevens-Woluwe, Belgium). Implant sites were prepared based on the bone density evaluated by the surgeon at the first drill. All the implants were inserted according to a one-stage protocol, with an insertion torque ranging from 35 to 45 N·cm. In case of poor bone density, the implant site was underprepared. Immediately after implant placement, patients of both groups received a digital impression (3M True Definition Scanner, 3M Italia, Pioltello, Milano) taken at implant level using dedicated abutments (Scan body type AQ, New Ancorvis srl), to check the position of the placed implants. Following implant placement, all patients received oral and written recommendations about the medication, oral hygiene maintenance, and diet. Implants were loaded after eight to 12 weeks of healing. Then, patients were followed twice a year for hygiene maintenance and occlusal control.

## 3. Outcome Measurements

Outcome measurements were implant failure, template-related complications, and accuracy.

An Implant was considered failed if it was lost for any reason (mobility, fracture or any infection). The stability of each individual implant was measured according to a previously published study. [7]. Fracture and/or infection were evaluated clinically and radiographically.

Any complications involving the surgical template was considered as template-related complication, including but not limited to, mismatching of the surgical template and fracture.

All the complications were recorded during at implant placement or during the follow-up by the same clinician (MT), who performed all the surgical procedures.

**Figure 4:**
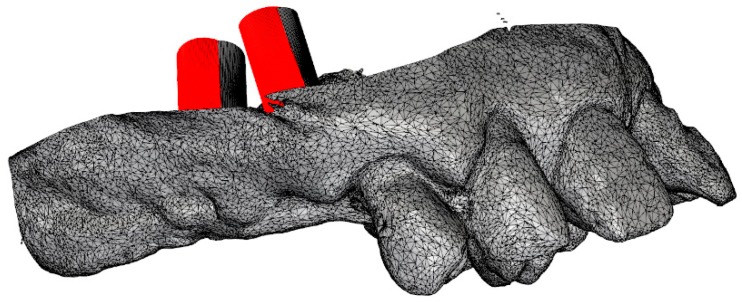
Superimposition of the Surface Tessellation Language (STL) derived from the planning with the STL taken after implant placement.

**Figure 5:**
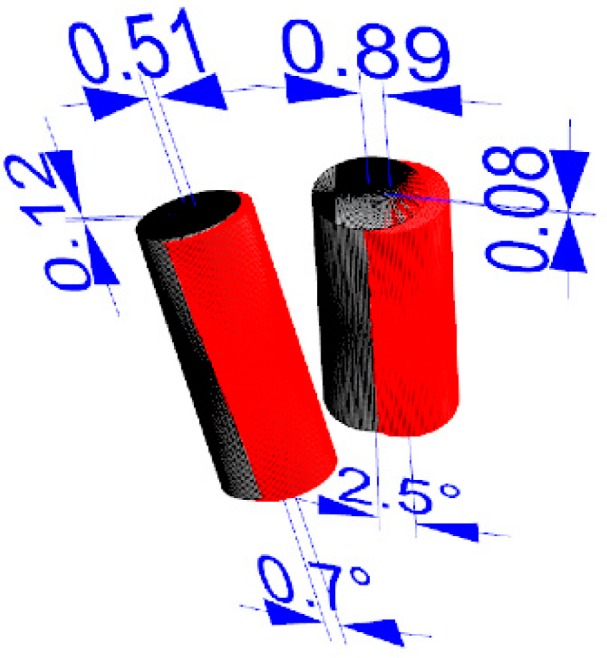
Measurement of the virtual planning accuracy by superimposition of the STL derived from the planning with the STL taken after implant placement.

Accuracy: Three deviation parameters (horizontal, vertical, and angular) were defined and calculated between the planned and placed implant positions according to a previously published study [7]. An expert mechanical engineer (FC) not previously involved in the study, performed all the measurements (Figure 4 and Figure 5).

## 4. Randomization

One computer-generated restricted randomization list was created by independent investigators, not involved in the selection and treatment of any patients. The random codes were enclosed in sequentially numbered, identical, opaque, sealed envelopes, that were opened consecutively, immediately after final approval of the computer-assisted plan.

## 5. Statistical Analysis

No “a priori” sample size calculation was made. Patient data were collected in a Numbers spreadsheet (Version 3.6.1 for Mac OS X 10.11.4). A bio-statistician analyzed the data using SPSS software for Mac OS X (version 22.0; SPSS Inc., Chicago, IL, USA) for statistical analysis. Descriptive analysis was performed for numeric parameters using mean ± standard deviation and median with confidence interval (95% CI). Implant failure and template-related complications between the two interventions were compared using the Fisher’s exact probability test. The nonparametric Mann-Whitney U test were used to compare the mean differences in horizontal, vertical, and angular deviation between groups. All statistical comparisons were conducted at the 0.05 level of significance.

## 6. Results

**Figure 6:**
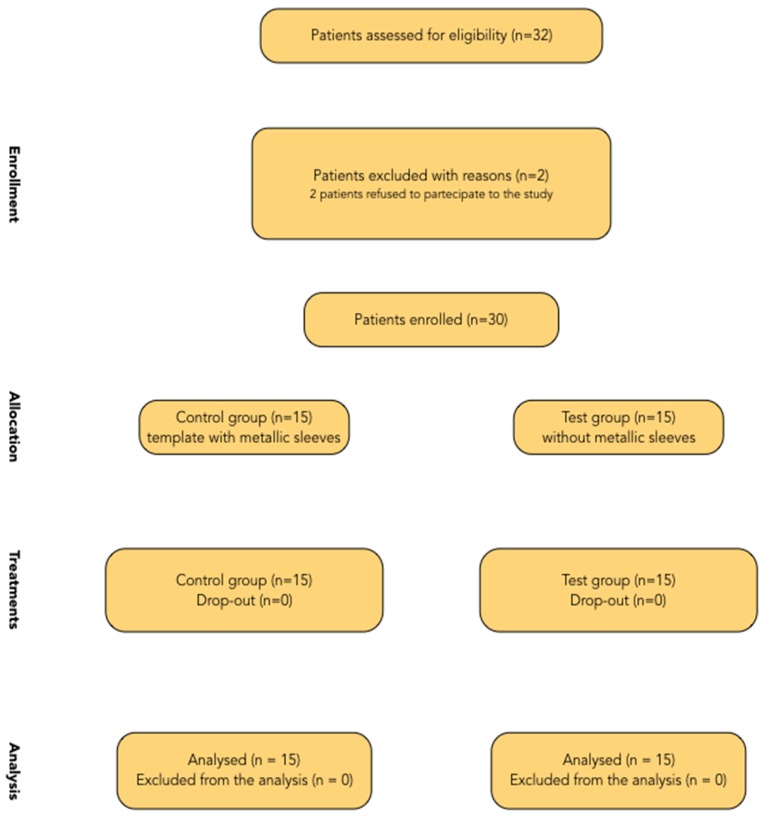
Consolidated Standards of Reporting Trials (CONSORT) diagram.

A flow diagram of activities through the phases of the trial is shown in Figure 6. Thirty-two patients were considered eligible for this trial. Two patients were not included, because they refused to participate in this study. No patient dropped out, and all patients were treated according to the allocated interventions.

Fifteen patients (eight female and seven males with a mean age of 45.1 years) were randomized to the control group (template with metallic sleeves) and 15 (10 female and five males with a mean age of 55.2 years) to the test group (without metallic sleeves). A total of 41 implants were placed in the control group while 49 implants were placed in the test group. Of these, 16 implants were placed through open sleeves and 33 through closed sleeves.

No implants failed and no complications were experienced at 6 months after loading follow-up. All the implants were inserted according to the manufacturer’s instructions, with an insertion torque ranged between 35 and 45 N·cm.

In the control group, the analysis of the final implant accuracy revealed a total mean error of 2.25 ± 1.41° (range 0.3–5.0°; 95% CI 0.52 to 1.65°) in angle; 0.52 ± 0.30 mm (range 0.1–1.1 mm; 95% CI 0.39 to 0.61 mm) in the horizontal plan (mesio-distal), and 0.58 ± 0.44 mm (range 0.0–1.6 mm; 95% CI 0.44 to 0.76 mm) in the vertical plan (apico-coronal). Overall, in the test group, the analysis of the final implant accuracy revealed a total mean error of 1.98 ± 2.38° (range 0.1–11.8°; 95% CI 0.13 to 1.47°) in angle; 0.61 ± 0.49 mm (range 0.05–2.53 mm; 95% CI 0.36 to 0.64 mm) in the horizontal plan (mesio-distal), and 0.37 ± 0.28 mm (range 0.0–1.3 mm; 95% CI 0.23 to 0.39 mm) in the vertical plan (apico-coronal). Comparing the mean value of the control group (closed metallic sleeves) with mean value of the closed sleeves of the test group, there was a statistically significant difference in angle (*p* = 0.0063) and in the vertical plan (*p* = 0.0126), with lower values in the test group. However, there was not a statistically significant difference in the horizontal plan (*p* = 0.7546) (Table 1).

Sub-group analysis relieved a mean error in angle of 3.3 ± 3.1° (range 0.2–11.8°; 95% CI 1.1 to 4.1°) with open sleeves and 1.35 ± 1.57° (range 0.1–5.9°; 95% CI 0.19 to 1.25°) with closed sleeves; the difference was statistically significant (*p* = 0.0268). In the horizontal plan (mesio-distal), the mean error was 0.87 ± 0.62 mm (range 0.2–2.53 mm; 95% CI 0.45 to 1.05 mm) with open sleeves and 0.51 ± 0.38 mm (range 0.05–1.7 mm; 95% CI 0.29 to 0.55 mm) with closed sleeves; the difference was slightly significant (*p* = 0.0477). In the vertical plan (apico-coronal), the mean error was 0.42 ± 0.33 mm (range 0.0–1.0 mm; 95% CI 0.19 to 0.51°) with open sleeves and 0.32 ± 0.24 mm (range 0.05–1.3 mm; 95% CI 0.22 to 0.38 mm) with closed sleeves; the difference was not statistically significant (*p* = 0.2929) (Table 2).

## 7. Discussion

In regards to the accuracy of the digital guided implant surgery, several works have been published in recent years with the aim to scientifically assess the accurateness of these techniques [8].

This randomized controlled trial was conducted with the aim to understand which kind of surgical template could be preferable to rehabilitate partial edentulous patients between CAD/CAM stereolithographic surgical guide with or without metallic sleeves. Furthermore, to evaluate possible differences in accuracy between open versus closed sleeves in case of sleeve-designed templates.

**Figure 7:**
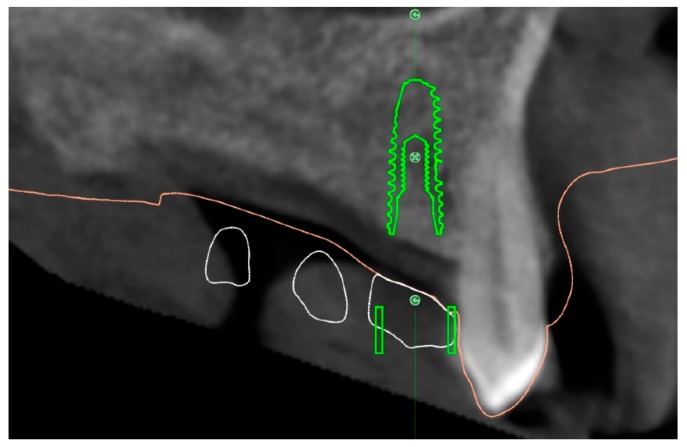
Collision between the mesial side of the metallic sleeve and the patient’s anatomy.

Both templates were able to achieve successful results, nevertheless, the null hypothesis that there would be no differences in implant accuracy between templates with or without metallic sleeves was partially rejected in favor of the hypothesis of differences. In fact, there was not a statistically significant difference in the horizontal position between the virtual and planned implant position, but there were statistically significant differences in angle and vertical discrepancy between the groups, with more accurate values for tubeless templates. A possible explanation is that the holes within the templates without metallic sleeves can be customized compared with standard metallic sleeves. On the contrary, the metallic sleeves cannot be modified in case of collision with soft or hard tissues (Figure 7). Hence, a flap must be elevated to avoid misfitting of the surgical template during its insertion. Otherwise, templates without metallic sleeves can have a customized fitting to the patient’s anatomy, reducing the risk of losing accuracy.

**Figure 8:**
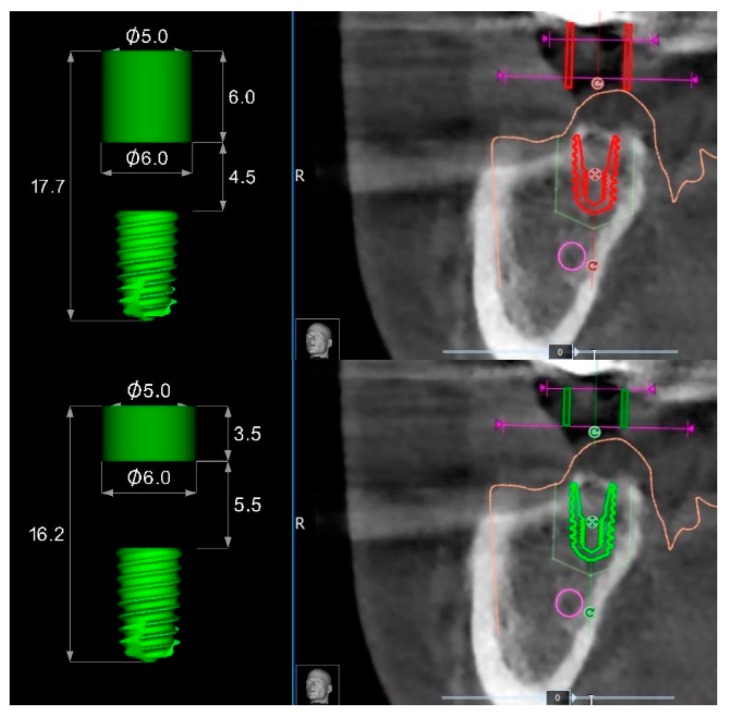
Differences between surgical templates with (lower) and without (upper) metallic sleeves for a regular diameter implant of 7 mm length.

Accordingly, in the templates without metallic sleeves, the contact points between the template and the surgical drills with the entire space between the soft tissue and the templets results in a longer guide compared to the 3.5 mm of standard metallic sleeves, plus 1 mm of the metallic reduction (Figure 8) in case of templates with metallic sleeves. This difference should allow for larger and more stable contact between the surgical template and the guided drills during the implant site development, slightly reducing the overall wobbling of the guided drills. Indeed, a longer guiding channel was found to reduce the angular deviations of implants in an in vitro investigation [9]. Lastly, the implant mount drivers used in combination with surgical templates designed without metallic sleeves (NoMount Driver and Fixture Driver, OneGuide Kit, Osstem) are designed without any stop drill to the surgical guide. A stop drill should touch the surgical template on the side, creating a high pressure on the template that could generate a distortion of the same template, resulting in a reduction of the final implant accuracy [7]. Another benefit of the surgical templates designed without metallic sleeves was the reduced mesio-distal space. In fact, the metallic tubes have a thickness of 0.5 mm, which could make it impossible for their use in the case of narrow mesio-distal distance, such as in case of lower incisor or upper lateral incisors. In this case, the manual insertion of the implant after removing the surgical template is required.

To the best of our knowledge, at the time of writing this article, this was the only RCT comparing the accuracy of the surgical template with and without metallic sleeves. This makes it difficult to evaluate how the present results were consistent with other comparable studies. Nevertheless, recent independent studies reported similar results in three-dimensional deviation between virtual planning and final implant positions [10,11,12].

In the last years, high precise 3D printing machines have become popular in dentistry. Among their applications, 3D printing machines can be used for surgical guide production, using biocompatible acrylate materials. Surgical templates without metallic sleeves can be easily designed and customized using dedicated software. This allows us to eliminate the metal tubes, and possibility to reduce the tolerance between the printed sleeves and the drill-guiding keys [13].

In the present study, closed sleeves showed higher accuracy in the angle compared to the open holes. According to reference [2], the maximum acceptable value for angle discrepancy should range between 5.9 and 16.7° depending on the implant length and diameter. In the present study, a mean discrepancy of 2.89 degrees was experienced. It should not involve clinical results because it is under the safety margin of the virtual planning. Furthermore, the clinicians can choose between open or closed sleeves, limiting the use of the open holes only in case with limited access in the posterior areas. In fact, according to previous reports, limited access is one of the most experienced complications when a surgical template was used [10,14], not allowing for a guided approach.

In an in vitro study by Schneider et al. it was shown that the tolerance of surgical instruments and therefore the amount of lateral movement of drills can be significantly reduced by using a modified protocol for surgical guide production. This protocol includes CAD and the use of 3D printing for surgical guide production without the use of any metal sleeves and with a more intimate contact between the guide and the drill-guiding drill key [13].

Despite the evident benefits related to higher accuracy in the vertical plan and angle experienced with the tubeless templates, there is still the need to clinically evaluate the long-term aesthetic and functional advantages. Particularly, it would be useful in the future to determine whether possible wear of sleeve-designed templates may affect the final implant position. The main limitation of the present study was the small sample size. Nevertheless, this limitation can only be solved by performing more similar trials with larger sample sizes, calculated based on these preliminary results.

The positive side is that this RCT is the only one published to date that makes such a comparison. Therefore, we hope that with this study as a precedent, other researchers will be stimulated to test similar hypotheses. Such studies are difficult to conduct for the reasons previously mentioned but are badly needed to understand the correct approach when dealing with teeth with an uncertain prognosis.

## 8. Conclusions

With the limitation of the present randomized controlled trial, the surgical templates designed without metallic sleeves were more accurate in the vertical plan and angle compared to the conventional template with metallic sleeves. Open sleeves should be used with caution in the molar region only in case of reduced interarch space. Nevertheless, in both groups, the maximum tridimensional deviations (angular, horizontal, vertical) did not exceed the safe offset of the software. Further research with a higher sample size and longer follow-up are needed to confirm these preliminary results.

## Figures and Tables

**Table 1:** Analysis of the final implant accuracy.

	Control Group	Test Group
Angle (°)	2.25 ± 1.41° (range 0.3–5.0°; 95% CI 0.52 to 1.65°)	1.98 ± 2.38° (range 0.1–11.8°; 95% CI 0.13 to 1.47°)
Horizontal plan (mm)	0.52 ± 0.30 mm (range 0.1–1.1 mm; 95% CI 0.39 to 0.61 mm)	0.61 ± 0.49 mm (range 0.05–2.53 mm; 95% CI 0.36 to 0.64 mm)
Vertical plan (mm)	0.58 ± 0.44 mm (range 0.0–1.6 mm; 95% CI 0.44 to 0.76 mm)	0.37 ± 0.28 mm (range 0.0–1.3 mm; 95% CI 0.23 to 0.39 mm)

**Table 2:** Sub-group analysis of the final implant accuracy.

	Open Sleeves	Closed Sleeves
Angle (°)	3.3 ± 3.1° (range 0.2–11.8°; 95% CI 1.1 to 4.1°)	1.35 ± 1.57° (range 0.1–5.9°; 95% CI 0.19 to 1.25°)
Horizontal plan (mm)	0.87 ± 0.62 mm (range 0.2–2.53 mm; 95% CI 0.45 to 1.05 mm)	0.51 ± 0.38 mm (range 0.05–1.7 mm; 95% CI 0.29 to 0.55 mm)
Vertical plan (mm)	0.42 ± 0.33 mm (range 0.0–1.0 mm; 95% CI 0.19 to 0.51°)	0.32 ± 0.24 mm (range 0.05–1.3 mm; 95% CI 0.22 to 0.38 mm)
